# Genetic Counseling and Management: The First Study to Report NIPT Findings in a Romanian Population

**DOI:** 10.3390/medicina58010079

**Published:** 2022-01-05

**Authors:** Cristina Gug, Ioana Mozos, Adrian Ratiu, Anca Tudor, Eusebiu Vlad Gorduza, Lavinia Caba, Miruna Gug, Catalina Cojocariu, Cristian Furau, Gheorghe Furau, Monica Adriana Vaida, Dorina Stoicanescu

**Affiliations:** 1 Department of Microscopic Morphology, “Victor Babeş” University of Medicine and Pharmacy, 300041 Timisoara, Romania; dr.cristina.gug@gmail.com (C.G.); dstoicanescu@gmail.com (D.S.); 2 Department of Functional Sciences, Discipline of Pathophysiology, “Victor Babes” University of Medicine and Pharmacy, 300173 Timisoara, Romania; 3 Center for Translational Research and Systems Medicine, “Victor Babes” University of Medicine and Pharmacy, 300173 Timisoara, Romania; 4 Department of Obstetrics and Gynecology II, “Victor Babes” University of Medicine and Pharmacy, 300041 Timisoara, Romania; dr.ratiu@gmail.com; 5 Obstetrics-Gynecology Clinic IV, Municipal Emergency Clinical Hospital, 300231 Timisoara, Romania; 6 Department of Functional Sciences, Discipline of Medical Informatics and Biostatistics, “Victor Babes” University of Medicine and Pharmacy, 300041 Timisoara, Romania; anca.ancutza@gmail.com; 7 Department of Medical Genetics, Faculty of Medicine, “Grigore T. Popa” University of Medicine and Pharmacy, 700115 Iasi, Romania; vgord@mail.com (E.V.G.); lavinia_zanet@yahoo.com (L.C.); 8 Faculty of Medicine, “Victor Babeş” University of Medicine and Pharmacy, 300041 Timisoara, Romania; miruna.gug@gmail.com (M.G.); cati.cojocariu95@gmail.com (C.C.); 9 Life Sciences Department, Faculty of Medicine, “Vasile Goldis“ Western University of Arad, 310414 Arad, Romania; cristianfurau@gmail.com; 10 General Medicine Department, Faculty of Medicine, “Vasile Goldis“ Western University of Arad, 310414 Arad, Romania; gfurau@yahoo.com; 11 Department of Anatomy and Embryology, “Victor Babes” University of Medicine and Pharmacy, 300041 Timisoara, Romania; vaida.monica@umft.ro

**Keywords:** prenatal diagnostics, prenatal screening, next-generation sequencing, non-invasive prenatal testing

## Abstract

*Background and Objectives*: Non-invasive prenatal testing (NIPT) has been confirmed as the most accurate screening test for trisomies 21, 18, 13, sex chromosomes aneuploidies and several microdeletions. This study aimed to assess the accuracy of cell free DNA testing based on low-level whole-genome sequencing to screen for these chromosomal abnormalities and to evaluate the clinical performance of NIPT. *Materials and Methods*: 380 consecutive cases from a single genetic center, from Western Romania were included in this retrospective study. Cell-free nucleic acid extraction from maternal blood, DNA sequencing and analysis of sequenced regions were performed by BGI Hong Kong and Invitae USA to determine the risk of specific fetal chromosomal abnormalities. In high-risk cases the results were checked by direct analysis of fetal cells obtained by invasive methods: 6 chorionic villus sampling and 10 amniocenteses followed by combinations of QF-PCR, karyotyping and aCGH. *Results:* NIPT results indicated low risk in 95.76% of cases and high risk in 4.23%. Seven aneuploidies and one microdeletion were confirmed, the other results were found to be a false-positive. A gestational age of up to 22 weeks had no influence on fetal fraction. There were no significant differences in fetal fraction across the high and low risk groups. *Conclusions:* This is the first study in Romania to report the NIPT results. The confirmation rate was higher for autosomal aneuploidies compared to sex chromosome aneuploidies and microdeletions. All cases at risk for trisomy 21 were confirmed. Only one large fetal microdeletion detected by NIPT has been confirmed. False positive NIPT results, not confirmed by invasive methods, led to the decision to continue the pregnancy. The main limitation of the study is the small number of patients included. NIPT can be used as a screening method for all pregnancies, but in high-risk cases, an invasive confirmation test was performed.

## 1. Introduction

Non-invasive prenatal testing (NIPT) has been increasingly used in the last decade, initially for the screening of common fetal aneuploidies and then of several structural chromosomal abnormalities [1,2,3]. Conventional aneuploidy screening technology was introduced in 2011. The discovery of cell-free fetal DNA in the plasma of pregnant women by Lo et al. in 1997 [4] provided the opportunity for NIPT. The introduction and rapid progress of next generation sequencing (NGS) technologies have increased the accuracy of the method [5]. Several studies reported a combined specificity for aneuploidies of 99.9%, much more accurate than biochemical screening [6,7,8,9,10]. Sonography and maternal biochemistry prenatal screening of fetal aneuploidies have a detection rate within 50–95% [5].

Sex chromosome aneuploidies that can also be identified using NIPT have a lower detection rate, especially monosomy X, with a higher false positive rate compared to autosomal trisomies. The average positive predictive value (PPV) for sex chromosome anomalies screening was estimated at 40.56% [5,11]. NIPT has several limitations regarding the detection of more rare fetal chromosomal abnormalities, other than aneuploidy for 3 (13, 18, 21) or 6 pairs (13, 18, 21, 9, 16, 22) of autosomal chromosomes, or the identification of cases with mosaicism.

Segmental duplications or deletions, also called copy number variations (CNVs), are extensively distributed in the human genome and many are associated with diseases [12,13,14,15,16,17]. The incidence of microdeletions/microduplications is independent of maternal age [18]. Structural chromosome abnormalities also remain difficult to detect. An algorithm known as Fetal Copy Number Analysis through Maternal Plasma Sequencing was developed to detect, close to 100%, deletions/duplications ≥10 Mb [19].

NIPT is widely used today, it is especially applied for the screening of autosomal trisomies, with the advantage of not requiring invasive sampling and having high accuracy, sensitivity and specificity [20]. Based on multiple meta-analyses, false positive rates range between 0.04% and 0.06% for the autosomal trisomies 21, 18 and 13. The detection rate of sex chromosome aneuploidies varies, being 90.3% for monosomy X with a 0.23% false positive rate. The detection rate for sex chromosome trisomies was 93.0%, and the false positive rate was 0.14% [21]. Invasive diagnostic tests such as amniocentesis or chorionic villus sampling remain the gold standard for the accurate diagnosis of chromosomal abnormalities.

In the present study, we aimed to assess the accuracy of cell free DNA (cfDNA) testing based on low-level whole-genome sequencing to screen for common aneuploidies and some microdeletions and evaluated the clinical performance of NIPT using maternal plasma cfDNA.

## 2. Materials and Methods

### 2.1. Patients and Sample Collection

A retrospective study was conducted including data obtained from a single private genetic center that collects cases from the western part of Romania. From September 2014 to September 2020, the NIPT was performed in 380 Caucasian pregnant women, including 4 twin pregnancies and 7 pregnancies obtained by in vitro fertilization (IVF). Exclusion criteria were as follows: more than two fetuses, pregnancy after stem cell therapy or organ transplantation, invasive cancers. All pregnancies were assessed by the obstetrician–gynecologists before maternal blood sampling. All subjects had a pretest ultrasound scan to determine the gestational age and number of fetuses.

The study was conducted in accordance with the Declaration of Helsinki. Ethical approval was provided by the Human Research Ethics Committee from the Emergency Clinical Municipal Hospital Timisoara, Romania (No. 6-2846 from 8 July 2020). Written informed consent was obtained from all investigated women to perform NIPT and to publish the results in the future.

Two types of tests, NIFTY (Non-Invasive Fetal Trisomy) in 225 cases and NIPS (noninvasive prenatal screening) in 155 cases were used. The samples were processed in Hong Kong and the USA, respectively. The NIFTY test was the first NIPT available in Romania. To our knowledge, NIPS has a very good accuracy-cost ratio (in our country NIPT is not covered by health insurance).

### 2.2. Genetic Counseling

Genetic counseling was provided two or three times to each couple. Maternal age, gestational age, *biochemical* or ultrasound markers results, obstetric history, and the existence of genetic diseases in the family were recorded during pretesting counseling, and the pedigree analysis was performed. The second genetic counseling was performed after the genetic screening using NIPT. The future parents were informed about the result of the test, which showed a low or high-risk of chromosomal abnormalities. In all high-risk cases, we recommended and performed a genetic diagnosis using an invasive method, chorionic villus sampling (CVS), or amniocentesis, followed by a combination of tests: Quantitative Fluorescent Polymerase Chain Reaction (QF-PCR), array Comparative Genomic Hybridization (aCGH) and karyotyping. After obtaining the result, a third genetic counseling session was performed, in order to explain the significance of the obtained results.

### 2.3. NIPT: NIFTY and NIPS Tests

The NIFTY test (BGI Laboratory) enables the isolation of cfDNA (including both maternal and fetal DNA) from a maternal blood sample and performs low coverage whole genome sequencing using NGS technology. The unique reads for each chromosome are calculated and compared to an optimal reference control sample. Data is analyzed using a BGI’s bioinformatics algorithm, and a risk score is generated for the tested conditions. For gender identification, cfDNA isolation is necessary (including both maternal and fetal DNA) from a maternal blood sample, followed by molecular genetic testing.

The NIFTY test enables the identification of 6 autosomal aneuploidies (chromosomes 13, 18, 21, 9, 16, and 22), sex chromosomes aneuploidies and 84 microdeletion syndromes. The test was validated for deletion/duplication (del/dup) syndromes larger than 10 Mb. The detection rate is over 90% if the size of the deletion/duplication is 3 Mb, according to the specifications of the BGI Laboratory. The NIFTY test was used during 2014–2020 in 225 cases.

The other test we used was NIPS, which allows the identification of pregnancies at risk for aneuploidies of chromosomes 13, 18, 21, and for 6 microdeletion syndromes: 1p36 deletion syndrome, 4p16.3 (Wolf syndrome), 5p15.2 (Lejeune syndrome), 15q11.2 (Prader-Willi and Angelman syndromes) and del (22q11.2) in singleton pregnancies. Invitae laboratory uses cfDNA extraction from maternal blood, DNA sequencing and analysis of sequenced regions to determine the risk of the above mentioned fetal chromosomal abnormalities. Other mechanisms that cause Prader-Willi and Angelman syndromes, such as uniparental disomy, have not been examined. The NIPS test was used during 2019–2020 on 155 cases. A percentage of Y chromosome ≥ 0.0048 and <0.0048 is considered as a male and a female fetus, respectively. The accuracy is at least 97%.

The NIPT is validated to detect fetal chromosome trisomy 21, 18, and 13 and the presence of the Y chromosome for twin pregnancies at a minimum gestational age of 10 weeks 0 days. Both tests can reveal fetal sex and sex chromosomes aneuploidies.

### 2.4. Cell Culture

An invasive method for fetal cell sampling (CVS or amniocentesis) was performed in every case with positive NIPT results. The invasive diagnostic procedures were performed by the obstetricians. All cytogenetic analyses were performed in our laboratory. Amniotic or chorionic villus cell cultures were initiated in 2 different flasks with AmnioMAX™-II complete medium (Gibco-Thermo Fisher, Waltham, MA, USA) containing fetal bovine serum, gentamicin and L-glutamine. On the fifth day of cell culture, the growth medium was changed, and subsequently, cell growth and number of mitoses were monitored daily. Long-term cultures were performed (12 days), and the cells were then harvested by routine techniques.

### 2.5. Cytogenetics

At least 20 metaphases per each sample were analyzed using GTG banding (at the 550-band level). Results were regularly available within 14–18 days. Analysis of the chromosomes was performed using LUCIA Karyo G software, and the aberrations and karyotypes were classified according to the International System for Human Cytogenomic Nomenclature 2016 [ISCN, 2016].

### 2.6. QF-PCR Assay

If a high-risk of aneuploidy for chromosomes 13, 18, 21, X, and Y was detected by NIPT/NIPS, the samples were verified by the rapid QF-PCR assay. IVD QF-PCR Devyser (Devyser AB, Stockholm, Sweden) and Elucigene (Tepnel Diagnostics, Manchester, England) kits were used. Promega Wizard™ Genomic (Promega, Madison, WI, USA) kit was used for DNA purification. Commercially available kits were used for the quantitative PCR reaction and ABI3730xl platform (Applied Biosystems, Waltham, MA, USA) was used for the amplicons migration. QF-PCR tests were performed by a partner laboratory (CytoGenomic Medical Laboratory, Bucharest, Romania).

### 2.7. Array CGH

Array CGH was performed using Cyto Chip Focus Constitutional BAC array (Illumina Inc., San Diego, CA, USA), and the used reference was the normal human male genomic DNA (Promega Corporation, Madison, WI, USA). Array CGH analyses were also performed by CytoGenomic Medical Laboratory.

### 2.8. Data Analysis

For a statistical presentation, the results were reconsidered after classifying pregnancies according to maternal age (<34 years and ≥35 years).

Statistical processing was performed using SPSSv17 (version 17, SPSS Inc., Chicago, IL, USA) and Microsoft Excel (version 2013, MS Corp., Redmond, WA, USA). In the case of nominal variables, the frequency tables were prepared together with the “pie” type graphs and the associations between these types of variables were performed with the Chi^2^ test; also, for some of these variables we made Risk Analysis (Odds ratio calculated with the 95% confidence interval for OR). For the numerical variables, the descriptive statistics were calculated, and histograms and column graphs were made. Comparisons between numerical series were performed with nonparametric tests because our continuous variables had non-Gaussian distribution; in case of comparisons between two series of values we applied the Mann-Whitney U test in case of comparisons between two series of values with non-Gaussian distribution and with Kruskal-Wallis test for comparisons between more than two series. The correlations between two numerical series were made with Spearman’s correlation analysis. We used the value of *p* < 0.05 for significant differences.

We calculated positive predictive value (PPV) and negative predictive value (NPV) both for aneuploidies and microdeletions, as we had confirmed cases, but also false positive results. PPV is defined as the probability that a positive result will be true and NPV is the probability that a negative result will be true. Public databases (OMIM, DECIPHER, NCBI, ClinVar) were used to interpret the data.

## 3. Results

### 3.1. Distribution of Cases by Years, Depending on the Identified Anomalies

**Figure 1:**
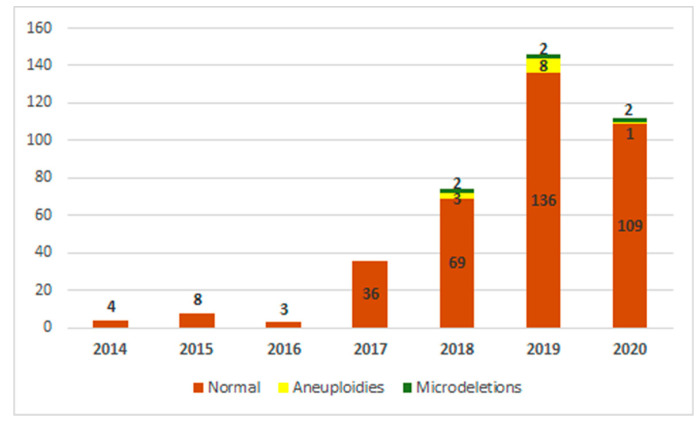
Distribution of cases over time, specifying the type and number of detected abnormalities (aneuploidies and microdeletions).

From September 2014 to September 2020, NIPT was performed in 380 pregnant women in a private genetic center from Timisoara, Romania. There were 380 cfDNA maternal plasma samples and 378 of them were successful (99.47%). In two cases, the analysis could not be performed even after a second blood sample was taken. Seven pregnancies resulting from IVF were included. A low risk was identified in 95.76% (362/378) of cases that were considered normal pregnancies and were further monitored. A high risk of aneuploidy has been identified in 2.64% (10/378) of cases. Increased risk for microdeletion syndromes has been identified in 1.05% (4/378) of cases. Combined anomalies risk (one aneuploidy and one microdeletion) has been identified in 0.52% (2/378) of cases. The number of investigations increased from 2014 to 2020, which shows the growing interest in this type of testing for pregnant women in Romania. Figure 1 shows the distribution of cases and anomalies identified throughout the studied period.

### 3.2. Analysis Duration for the NIPT

Given that the tests were performed in Hong Kong or in the USA, and that blood samples had to be sent by international transport, part of the time was “lost”. However, the average duration of the analysis was 7 days, ranging from 4 to 10 days.

### 3.3. Gestational Age at the Time of NIPT

**Figure 2:**
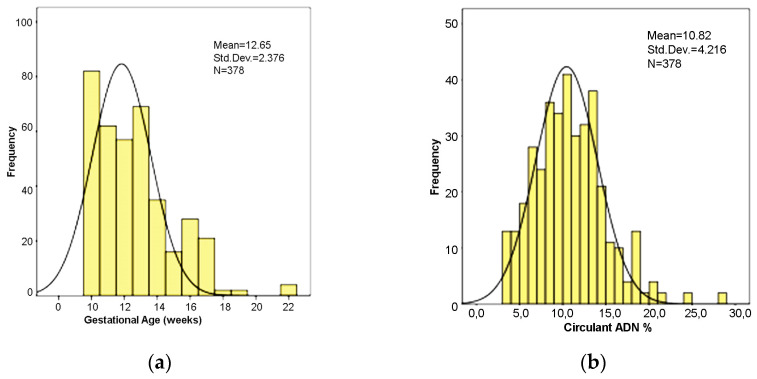
(**a**) Distribution of gestational age values. (**b**) Distribution of cell-free DNA percentage in maternal blood.

The mean gestational age was 12.62 ± 2.47 weeks (range: 10–22 weeks) (Figure 2a). Most cases (55 cases) had a gestational age of 10 weeks.

### 3.4. Fetal Fraction

NIPT is based on the percentage (%) of cell-free DNA in the mother’s blood, also called fetal fraction (FF). In our study, these percentages were highly variable, with a mean of 10.96% ± 4.14, ranging between 3.5% and 28.12% (Figure 2b).

In 4 cases the analysis failed initially, but it was successful in 2 of them after a second blood sample was taken, hence finally, 378 cases received a NIPT risk result. FFs in these 2 cases were 8.58% and 10.96%, respectively. As FF values were within such a wide range, we evaluated the relationships between FF and different parameters to find significant associations (Table 1).

A gestational age of up to 22 weeks had no influence on fetal fraction. We did not find significant differences in fetal fraction across the high and low-risk groups.

The results of the associations between FF and different parameters were not statistically significant (Table 2).

### 3.5. Sex of the Fetus

The identification of the Y chromosome indicated the male fetal sex. Fetal sex was female in 52.64% (199/378) of cases and male in 47.35% (179/378), with a sex ratio = M/F of 0.899. As the X and Y chromosomes can be identified, by default, sex chromosome aneuploidies can be identified as well. In this study group no such syndromes were identified, although two results indicated a high-risk of X monosomy, but this aneuploidy was not subsequently confirmed by the invasive method.

### 3.6. Maternal Age

The mean maternal age in the entire group was 33.4 ± 4.96 years, ranging between 21 and 47 years. The patients were divided into two groups: under 34 years old and 35 years or older. We also calculated the mean age of pregnant women who were at risk for aneuploidy depending on the type of aneuploidy (Table 3).

**Figure 3:**
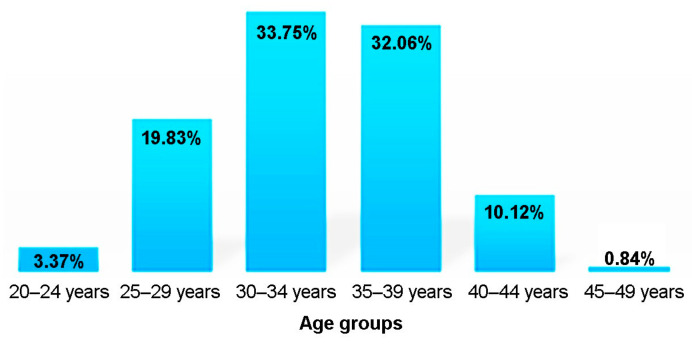
Distribution of cases considering maternal age.

The association between advanced maternal age risk (age > 35 years) and the aneuploidy risk was not statistically significant (chi-square test, *p* = 0.824). Age over 35 years was not a significant risk factor for the occurrence of aneuploidy (OR = 1.18, cu 95% CI = [0.5, 2.78]), *p* = 0.824 (Chi2 Test). Most cases were in the 30–34 age group, followed by the 35–39 age group (Figure 3).

### 3.7. The Main Indications for the NIPT

**Figure 4:**
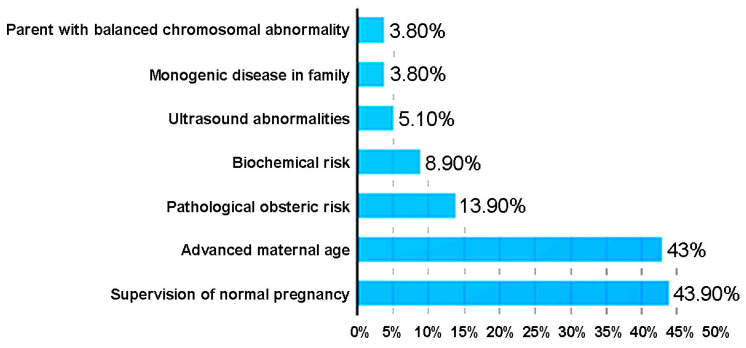
Indications for the NIPT.

The indications for non-invasive prenatal testing were established for all 380 patients during the pre-testing genetic counseling (Figure 4). Most of the tests were performed at the gynecologist’s indication for supervision of normal pregnancy, followed by the cases with advanced maternal age and positive obstetric history. Of the 8 cases with aneuploidy confirmed by invasive tests, 2 cases were in the age group under 34 years: maternal age was 25 years and trisomy 21 was confirmed in one case; another 30-year-old patient with twin pregnancy had a female twin with trisomy 18. There were also cases with biochemical risk at the double test (DT), prenatal ultrasound abnormalities or with a balanced chromosomal abnormality. A total of 3.8% of cases had a positive family history for single-gene diseases; in these latter cases, the test was performed to identify the sex of the fetus.

Ultrasound abnormalities were identified in 19 fetuses (5%), including 5 cases with increased fetal nuchal translucency (NT) (≥3 mm). The following abnormalities were recorded: intrauterine growth retardation, cardiac anomalies (tricuspid valve regurgitation, tetralogy of Fallot; aortic override, pulmonary artery stenosis, atrial septal defect), renal abnormalities (renal pyelectasis, bilateral hyperechogenic kidney), hyperechogenic fetal bowel, abnormal anterior abdominal wall, central nervous system abnormalities (banana-shaped cerebellum with risk of neural tube defect, choroid plexus cysts of 5 mm), abnormal venous duct with absent, bilateral hydrothorax, ascites, cystic hygroma, sexual ambiguity, polydactyly, clinodactyly. Combinations of indications for testing were registered in 12 cases (3.15%), one of them having 3 and another one having 4 indications (Supplementary Material: Table S1).

Four pregnant women (1.05%), carriers of an X-linked recessive gene, were offered the analysis for fetal sex determination. In all cases, there was a female fetus and the NIPT result was normal.

In 10 cases (2.63%), there was an indication related to the existence of a chromosomal disease in the family. In 6 cases there was a relative with trisomy 21. In 3 cases other chromosomal abnormalities were identified: maternal translocation, paternal inversion and a de novo duplication of a previous child.

### 3.8. The NIPT Results

Of the 378 cases, 362 (95.76%) had a low-risk, and 16 (4.24%) had a high-risk. Of these, 8 cases (2.12%) were confirmed by invasive methods, and 8 (2.12%) were false-positive.

Correlations between the risk assessed by NIPT and the results of invasive prenatal diagnosis have also been performed (Table 4).

In one case, the NIPT result suggested a large chromosomal deletion that was confirmed by aCGH, but with increased size, 61.88 Mb, compared to the size of 54.27 Mb determined by the NIPT. In this case, FF was 15.64%, the highest in the group at risk of microdeletions. The rest of the cases found to be at risk of microdeletions were not confirmed by direct methods (aCGH) (Table 5).

### 3.9. Invasive Methods for Verifying (Confirmation/Exclusion) NIPT Results

Direct analysis of fetal cells obtained by invasive methods was performed to verify the 16 cases identified to be at risk. Six CVS and ten amniocenteses were performed, followed by combinations of QF-PCR, karyotyping and aCGH. Thus, 29 analyzes were performed, of which 12 were QF-PCR, 11 karyotypes and 6 microarrays. One of the twin pregnancies benefited from 2 karyotypes and the other from 2 QF-PCR analyzes and 2 karyotypes.

In all cases at risk of aneuploidies, 12 fetal karyotypes and 10 QF-PCR analyzes were performed, the latter in order to obtain rapid results. The cases at risk of X monosomy and trisomy 14 were not confirmed.

Two cases at risk of aneuploidy, one with trisomy 21 and one with trisomy 18, were from twin pregnancies. Two amniocenteses were performed separately for each fetus, confirming trisomy 21 in the male fetus from one twin pregnancy and trisomy 18 in the female fetus from the other twin pregnancy.

Two cases were at risk for both numerical and structural abnormalities, and amniocentesis was required, followed by fetal karyotyping and microarray. One case (0.26%) was at risk for trisomy 14, which has not been confirmed, in association with Xp microdeletion, which was confirmed, and the pregnancy was stopped for medical reasons. Another case had a high-risk for trisomy 13 and for microdeletion 1p36, but they were both ruled out and the pregnancy continued.

A high-risk of microdeletion syndromes as unique abnormalities was identified in 4 cases (1.04%): del (10q25), del(15q), del(20q), and del(22q11.2). As mentioned, a high-risk for a microdeletion and an aneuploidy was identified in 2 cases: del(1p36) and trisomy 13 in one case and del(Xp) and trisomy 14 in the other one (Table 4 and Table 5). In all these 6 cases aCGH was performed. The case at risk for a large deletion, of 61.88Mb, del(X)(p22.33–11.21) was confirmed, but the other 5 cases proved to be a false-positive: del(10q25.2–q26.3), del(15)(q11.2–13.1), del(20q11.21–q13.13), and two microdeletion syndromes: 1p36 and del(22q11.2).

### 3.10. Positive Predictive Value (PPV)

Regarding the high-risk for aneuploidies, out of the 12 cases, 7 were confirmed; therefore, PPV was 58.33%, while regarding the high-risk for microdeletions, out of the 6 cases, only one was confirmed, and PPV was 16.66. Of the 12 aneuploidies, 5 cases (including one from a twin pregnancy) had an increased risk for trisomy 21, and all were confirmed; therefore, we report 100% PPV for trisomy 21. The other 3 cases (including one from a twin pregnancy) had an increased risk for trisomy 18, and two were confirmed; therefore, we report 66.66% PPV for trisomy 18. One case had an increased risk for trisomy 13, but it was ruled out, and we reported 0% PPV for trisomy 13. A special case was one obtained by IVF with an increased risk for X monosomy. CVS with QF-PCR ruled out this diagnosis, but identified trisomy 13, and post-test ultrasound at week 12 showed specific congenital anomalies (polydactyly). This case was a false-negative for trisomy 13 and false-positive for monosomy X. As it was the only case at high-risk for trisomy 13 in the entire group, the NPV was 100%. The second case with increased risk only for X monosomy has not been confirmed. Both cases at risk of X monosomy were false positive. A comparison of specificity, sensitivity, and PPV scores for aneuploidies is presented in Table 6.

## 4. Discussion

Until recently, prenatal screening for fetal aneuploidies relied on the measurement of maternal serum biochemical markers combined with fetal ultrasound markers [22]. At present, in Romania, NIPT is used as a commercial service in the clinical detection of common chromosomal aneuploidies and microdeletions. In Romania, in high-risk cases, invasive prenatal diagnosis became available in 2002 [23]. NIPT has become more widely accessible, giving women the opportunity to benefit from these services.

Non-invasive prenatal testing analyzing FF in maternal blood has rapidly developed in the last few years. It is used as a screening test for the most common chromosomal disorders. NIPT relies on the presence of fragments of cell-free placental and maternal DNA in maternal blood to assess the risk of an affected pregnancy. The test compares the total maternal and fetal DNA in a maternal blood sample to a control sample using advanced bioinformatics analysis. It is provided for screening only. False-negative and false-positive results may occur. This test assesses the risk for the following chromosomal abnormalities: trisomy 21, 18, 13, sex chromosome aneuploidies, and several microdeletions [19,24].

NIPT can be performed at any stage of the pregnancy, generally from 10 weeks’ gestation onwards, to ensure adequate cfDNA in the maternal plasma sample [25,26,27]. In our study group, NIPT was performed between 10 and 22 weeks of pregnancy. However, most cases came immediately after 10 weeks of gestation, with a mean of 12.62 ± 2.47 weeks.

### 4.1. Fetal Fraction

The proportion of cfDNA molecules is expressed as the fetal DNA fraction in the plasma of pregnant women [21]. We analyzed the association between FF and the following parameters: gestational age, maternal age, and NIPT risk result for aneuploidies and for microdeletions, but no significant correlations were found.

In our study, a gestational age of up to 22 weeks had no influence on FF, consistent with the results of Hestand et al. [28]. Other studies found that the FF positively correlated with gestational age [29] and significantly increased especially beyond 23 weeks of gestation, but waiting for a late gestational age is not a reliable approach [27,30].

We found lower FF values in pregnant women over 35 years compared to those under 35 years, but the difference was not statistically significant. Hou et al. [30] found that the percentage of FF significantly decreased with an increase in maternal age. A negative correlation between FF of cfDNA and maternal age was reported by Guo et al. [29], while Hestand et al. found no influence of maternal age on FF [28].

We did not find significantly increased FF values in pregnant women with low-risk NIPT results. In a study regarding FF in pregnancies with a low and high-risks for fetal chromosomal aneuploidies, Hudecova et al. reported no statistically significant difference in FF across the high, intermediate, and low-risk groups [31].

In our study, the FF values were not significantly increased in pregnant women at risk of a microdeletion. Using multivariate regression analysis to determine several maternal and fetal factors that might be significant predictors of the FF, Ashoor et al. found that fetal karyotype did not provide a significant independent prediction of FF [32]. Another study compared FF in euploid versus aneuploid pregnancies and found that the median FF was significantly higher in Down syndrome pregnancies and significantly lower in trisomy 18 and triploid pregnancies. Some results were not informative, and this was due to low FF, but the authors considered that the high-risk for trisomy 18 and/or triploidy warranted offering additional assessments [33]. NIPT could establish a risk in our study in 378 cases and the FF range was found between 3.5% and 28.12%, with a mean of 10.96 ± 4.14%. It was confirmed that the important prerequisites for NIPT are FF > 3.5% and gestational age over 10 weeks of pregnancy. An FF-based risk model was built for pregnancies with a FF too low to receive a result on standard NIPT. This algorithm can identify a subset of cases at increased risk for trisomy 13, trisomy 18, or triploidy [34].

Invasive assays used in our cases were CVS and amniocentesis. The main advantage of CVS is that it can be performed immediately after obtaining the NIPT result; hence a result can be obtained in the shortest possible time. Patients who underwent amniocentesis could not have CVS because the pregnancies were advanced, close to week 16.

### 4.2. Maternal Age

The mean maternal age in the group detected to be at high-risk for aneuploidies was 35.83 years (range between 25–44 years), but if we exclude the false-positive cases and consider only the confirmed aneuploidies, mean maternal age was 34.5 years (range between 25–42), very close to the mean age of the entire study group, which was 33.4 years (ranging between 21–47 years). The mean values were somewhat close, therefore not statistically significant, and considering the very wide ranges found in the study group, it did not help us in assessing the risk before NIPT was performed. This is one of the reasons why we advocate for performing the NIPT in all pregnancies, even in the absence of classical indications.

Pretest genetic counseling helps identify potential risks and often puts an order in the conflicting ideas of anxious pregnant women [35,36].

### 4.3. Aneuploidies

If a fetus has a trisomy, the fetal-derived DNA molecules from the extra chromosome should be increased in maternal plasma, when compared to a pregnancy with a euploid fetus [37,38].

Trisomy 21 was the only abnormality for which the NIPT risk result was confirmed in full, PPV being 100%. The mean maternal age in these cases was 34.8 years. According to BGI versus Invitae specifications for trisomy 21, sensitivity is 99.17% versus 99.99%, specificity is 99.95% versus 99.89%, and PPV is 92.19% versus 92.89% (Table 6). Only 20% of our cases had ultrasound abnormalities, 40% had abnormal prenatal biochemical screening results-double test, and another 40% had maternal age over 35 years as the only indication for NIPT. Yamada et al. reported a lower maternal age-specific risk for trisomy 21 based on the clinical performance of NIPT compared to the risk predicted in several other studies [39].

NIPT was positive for trisomy 18 in 3, cases but only 2 have been confirmed, PPV being 66.66%. Interestingly, the case with the false-positive result that was not confirmed by amniocentesis followed by QF-PCR was 44 years old. Much higher PPV values were available from both partner laboratories: 76.61% (BGI) and 89.11% (Invitae) (Table 6) [21,40,41].

One of our cases had a high-risk for trisomy 13 and for 1p36 microdeletion, both abnormalities being excluded after amniocentesis followed by QF-PCR and aCGH. The pregnant woman was 39 years old and gave birth to a healthy baby. In contrast, another 37-year-old woman with the pregnancy achieved by IVF, with cystic hygroma and heart abnormality on a prenatal ultrasound, and NIPT high-risk for monosomy X was found to have a fetus with trisomy 13 after CVS with QF-PCR was performed [42]. Preimplantation genetic testing was not performed in this case. Post-NIPT ultrasound identified polydactyly, and the pregnancy was stopped for medical reasons. In a report on NIPT using fetal DNA from maternal plasma for trisomy 13 detection, Yu et al. found the sensitivity and specificity of 95.2% and 99%, respectively [43].

NIPT identified an increased risk for monosomy X in two cases, but both were false-positive, PPV being 0% in our study group. Monosomy X has the lowest PPV, 40% according to BGI and 69.15% according to Invitae Laboratories [25,41].

Other trisomies can be detected by chance with the low genome coverage version. Of the aneuploidies found in our study group, only trisomy 14 falls into this group, noting that this abnormality is not part of the standard aneuploidies expected to be identified, but it is an abnormality that can be additionally detected by NIFTY [44]. In addition to the standard aneuploidies, in one case, a high-risk for trisomy 14 and for Xp deletion has been found. The 41-year-old pregnant woman had a positive medical history: a trisomy 21 pregnancy resulted in a stillbirth. The case was special because, after amniocentesis followed by QF-PCR, aCGH, and fetal karyotyping, trisomy 14 was ruled out; this was a false-positive result. The deletion of the short arm of the X chromosome was confirmed, and a translocation between chromosome 14 and the Xq arm was identified. The fetus had numerous ultrasound abnormalities, and termination of pregnancy for medical reasons was decided. Rare autosomal trisomies are important but not so rare. Scott et al. found that they are often associated with poor obstetric outcomes [45]. Preimplantation testing was performed in two pregnancies (2/7) obtained by IVF (outside Romania). NIPT was also carried out (NIFTY in one pregnancy and NIPS in the other one), confirming the sex of the fetus and the absence of aneuploidies.

### 4.4. Twins

Twin cases are particular in many ways. First, NIPT analysis can only evaluate the risk of aneuploidy and not of microdeletions. Our study included 2 pregnant women (30 and 36 years old) without other indications besides NIPT increased risk for trisomy 21 and trisomy 18, respectively. In both cases the twins were of different genders. In each case, two amniocenteses were performed followed by QF-PCR and fetal karyotyping and in both cases, one of the twins was confirmed with a trisomy (one male fetus with trisomy 21 and in the other pregnancy one female fetus with trisomy 18). In both cases, the parents decided to stop the pregnancy. Another peculiarity of twin pregnancies is related to the vanishing twin, whose sex can cause false-positive results for fetal gender. As the pregnancy progresses, the ultrasound sex is observed, and if there are discordances, a disorder of sex development must be considered. In these cases, genetic counseling and detailed anamnesis are especially useful and can further guide pregnancy management. BGI recommends waiting 8 weeks after the disappearance of a twin before taking the NIPT test; it is considered that the FF of the vanished twin has decreased considerably during these weeks and no longer influences the result. However, this is impossible to achieve when the pregnant woman presents to the first obstetric consultation too late for confirmation of pregnancy, a few weeks after the signs of a twin pregnancy disappears. Therefore, we encourage pregnant women to have their first obstetric consultation as soon as possible. A recent study has found that trisomy 21, 18, and 13, as well as X chromosome aneuploidies, were accurately detected by NIPT in twin pregnancies. As in our study, the aneuploidies mostly occurred in only one twin. The authors agreed that NIPT could be used as routine prenatal screening in twin pregnancies [46]. If there are more than two fetuses, the risk of a false-positive and negative results increases. In their study, Hartwig et al. reported four false negative aneuploidy cases, of which two were explained by a vanishing twin. The sensitivity and specificity, when no-calls and vanished twins were excluded, were 100% and 99.5% for trisomy 21, 91% and 99.2% for trisomy 18, and 100% and 99.6% for trisomy 13 [47].

Many factors, both maternal (such as maternal blood transfusion, surgery, immunotherapy, neoplasm, or mosaicism) and pregnancy-related (vanishing twin, fetal demise, confined placental mosaicism, true fetal mosaicism, uniparental disomy, and polyploidy), can affect the results. In our study, the pregnant women denied such medical conditions. Regarding pregnancy-related factors, fetal ultrasonography did not reveal the presence of a vanishing twin. The other above-mentioned chromosomal abnormalities were not identified with invasive techniques. Fetal demise during weeks 20–30 of gestation was registered in 2 cases, both having NIPT low-risk results, and the causes were not established. We also gathered information about births and offspring, revealing that a female newborn from a twin pregnancy was diagnosed with Treacher Collins syndrome after birth. All other newborns were considered healthy at birth without any suspicion of chromosomal syndromes.

### 4.5. Microdeletion/Microduplication Syndromes

Microdeletion and microduplication syndromes are caused by CNVs and are relatively rare disorders [48]. It is estimated that they account for 1–2% of all newborn congenital anomalies and the most common is 22q11.2 deletion syndrome [49].

NIPT test can detect specific loci relevant to microdeletion/microduplication syndromes according to OMIM and Decipher databases. The risk of false-positive/negative results in these cases can be increased compared to common trisomies. Due to the low prevalence of these microdeletion syndromes and the limited performance data for these disorders, it is not possible to calculate a precise and accurate PPV.

Our study included cases analyzed during 2014 using the NIFTY test. At that time, three aneuploidies (for chromosomes 13, 18, 21) and three microdeletions (1p36 deletion syndrome, 5p Cri du Chat syndrome, 2q33.1 deletion syndrome) could be tested. During 2015, the test was improved, enabling identification of six aneuploidies (chromosomes 9, 16, 22 were added) and eight microdeletion syndromes (5p, 1p36, Van der Woude 1q32.2, 2q33.1, 16p12.2, DiGeorge 2, Jacobsen 11q23, Prader-Willi/Angelman). In 2017 the number of microdeletions that could be tested increased to 63, and in 2018 the test changed its name to Nifty-pro, and the number of microdeletions increased to 84.

In our study, 5 out of 6 cases identified as having a high-risk of microdeletions, were ruled out; therefore our PPV was only 16.66%. One of our cases had a high-risk of Xp microdeletion, of 54.27 Mb; after aCGH was performed, it has been confirmed, but having a larger size, 61.88 Mb. FF, in this case, was 15.64%, the highest in the group at risk of microdeletions.

The other microdeletions, which were not confirmed, were smaller, their sizes ranging from 5.06 Mb to 22.57 Mb. The deletions identified in these 5 cases were: del (10q25), del (15q), del (20q), del 1p36 and del(22q11.2) microdeletion syndrome. All five were ruled out following direct analysis of fetal cells by aCGH. The gestational age was variable, ranging between 11–16 weeks.

Detection of microdeletions can be performed by the BGI laboratory through low coverage whole genome sequencing using NGS technology and a bioinformatics algorithm, which allows the identification of 84 deletions and duplications. Internal analysis of BGI showed a sensitivity exceeding 90% (cfDNA9.5%) in selected del/dup syndromes with abnormal size over 3 Mb.

The maternal presence of CNVs, as recently reported in two cases, could explain why the results yielded by NIPT are not confirmed by invasive methods [48]. Microdeletions can occur de novo in most cases but can also be inherited if a parent has a balanced translocation. Results should always be reviewed with a qualified healthcare professional. High-risk results must be followed by confirmatory diagnostic testing.

The PPV and NPV reported scores do not consider additional clinical information such as previous screening results, positive family history, or abnormal ultrasound findings. It is not possible to calculate a precise and accurate PPV for sex chromosome aneuploidies due to their uncertain prevalence and the limited performance data for these disorders. Moreover, due to the low prevalence of microdeletion syndromes and the limited performance data for these disorders, it is not possible to calculate a precise and accurate PPV as well. The Netherlands launched a study on NIPT as a first-tier test offered to all pregnant women. This study confirmed that genome-wide NIPT is a reliable and robust screening test. PPVs found by the authors were 96% for trisomy 21, 98% for trisomy 18, and 53% for trisomy 13 and were higher than expected [50].

Another study that examined over 8000 single pregnancies with NIPT reported that PPVs for trisomy 13, 18, 21 and sex chromosome aneuploidy were 14.28%, 60%, 80%, and 45.83%, respectively. At the same time, they have found 0.63% positive cases for chromosomal microdeletions or microduplications, but only 36.11% of them were true-positive cases. They concluded that NIPT had the highest accuracy for trisomy 21 detection, while accuracy was low for chromosomal microdeletion and microduplications [51].

### 4.6. Pre-Eclampsia

In one case, with low-risk NIPT, the fetal death was caused by pre-eclampsia, with onset at 25 weeks. Determining the genetic risk of hypertension before pregnancy is part of high-quality medical care because a proper low-salt diet can increase the chance that the pregnancy will progress to near term and give birth to a healthy baby. Various pathophysiological mechanisms have been involved in the onset of pre-eclampsia [52,53]. A comprehensive literature search of several databases conducted to identify relevant studies that evaluated cell-free fetal DNA levels in pregnant women before the clinical onset of pre-eclampsia revealed that cell-free fetal DNA quantification is a marker predicting the development of pre-eclampsia. The authors concluded that it could probably only be used from the beginning of the second trimester; otherwise, the detection rate is too low or not significant [54]. Several studies suggested that a low FF on NIPT might be correlated with future hypertensive disease [55,56,57], while Bender et al. found that FF at 10 to 20 weeks of pregnancy has not been associated with the development of gestational hypertension [58]. Even if there is a significant association between FF and first-trimester markers for adverse pregnancy outcomes, further research is needed to establish if it could act as an independent first-trimester marker in an algorithm for screening for pre-eclampsia [59].

### 4.7. Genetic Counseling

NIPT is an advanced screening test, incorporated into clinical practice in the context of genetic counseling. The genetic counselor should highlight that NIPT is a screening test, but the detection rate is superior to that of maternal serum screening, which has a misdiagnosis rate of 5 to 50% [8]. NIPT results may indicate an increased risk for specific conditions. The healthcare provider will further interpret the NIPT results in the context of the patient’s clinical data and family history and will recommend genetic counseling and additional testing when appropriate. Diagnostic testing should be performed to confirm a positive result [60].

In clinical practice, one patient requires several genetic counseling sessions [61]. This series of investigations with genetic counseling at each step requires a great involvement of the geneticist who coordinates the sequence of these tests and explains the results, sometimes inconsistent with the initial NIPT results.

In our study, four pregnant women (1.05%) were carriers of an X-linked recessive gene, and the analysis was performed for result-oriented care, focusing on the fetal sex. In all cases, the fetal gender was identified as female and had a low-risk NIPT. Pan et al. developed a fetal sex determination method based on maternal plasma sequencing and assessed its potential use in X-linked disorders counseling, concluding that high accuracy of non-invasive fetal sex determination can be achieved [62].

Several other cases had a history of single-gene diseases in the family, and even if NIPT could not detect a risk for these conditions, further explanations were needed, and amniocentesis was recommended. In two cases with advanced maternal age, after a low-risk NIPT result, the management of the pregnancy was oriented towards amniocentesis to test the fetus for known mutations that could have been inherited from the carrier’s parents [63,64,65,66].

NIPT newer technologies have already expanded prenatal screening beyond common autosomal aneuploidies to smaller chromosomal abnormalities. Other tests for single-gene disorders are currently developed, but their validation is important. Parents should be provided with appropriate genetic counseling by a qualified professional [67,68].

### 4.8. Strengths and Limitations

Any result that indicates a high-risk NIPT should be followed by an invasive method, but not all pregnant women accept it easily. In such cases, it is beneficial to explain that the invasive method remains the “gold standard” in diagnosing genetic diseases and clarifying diagnostic controversies. On the other hand, in cases with ultrasound abnormalities and a low-risk NIPT result, the area of investigations was widened in order to identify the cause and establish the management of the pregnancy.

Although one limitation of the study is the small sample size, the study is valuable because it is the first to present NIPT issues in a group of patients in Romania. We hope that sharing our experience will be useful to specialists working in this field. Another study limitation is related to the lower number of microdeletions that could be initially detected and which increased over time. Not all patients were tested for the same number of microdeletions. The study also reflects the progression over time of NIPT’s scientific knowledge and investigative power.

## 5. Conclusions

This is the first study in our country reporting the NIPT results from a series of 380 cases (Caucasians from Eastern Europe). In our study, confirmation rate was higher for autosomal aneuploidies compared to sex chromosome aneuploidies and microdeletions. All cases at risk for trisomy 21 were confirmed. A trisomy 13 with a false-negative NIPT result was identified via invasive prenatal diagnosis. Only one large fetal microdeletion detected by NIPT has been confirmed. False-positive NIPT results, which were not confirmed by invasive methods, led to the decision to continue the pregnancy, bringing relief to pregnant women. A gestational age of up to 22 weeks had no influence on fetal fraction. We did not find significant differences in fetal fraction across the high and low-risk groups. The main limitation of the study is the small number of patients included. NIPT can be used as a screening method for all pregnancies, but in high-risk cases, an invasive confirmation test is strongly recommended, and no definitive measure of pregnancy termination should be taken without a result obtained by an invasive method.

## Figures and Tables

**Table 1:** Fetal fraction descriptive statistics for different parameters.

Parameters	Result	Number of Cases	Mean FF ^1^	SD ^2^	Standard Error of the Mean
Maternal age	Up to 34 years	215	11.23	4.28	0.37
	Over 35 years	163	10.61	3.93	0.39
NIPT risk of aneuploidy	No risk	362	11.03	4.14	0.28
	Risk for aneuploidy as a unique abnormality	10	10.40	4.18	0.82
NIPT risk of aneuploidy NIPT risk of microdeletion	Risk for aneuploidy and microdeletion	2	10.21	4.07	0.54
	No risk	372	10.93	4.16	0.27
	Risk for microdeletion as a unique abnormality	4	12.57	2.76	1.23
NIPT risk of microdeletion Risk confirmation	Risk for microdeletion and aneuploidy	2	11.03	3.07	0.89
	Not necessary	362	11.00	4.22	0.28
	Not confirmed	7	9.17	2.40	0.98
Risk confirmation	Confirmed	9	11.24	2.61	0.87
	Total	378	10.96	4.14	0.27

FF ^1^, Fetal fraction (%); SD ^2^, standard deviation; NIPT ^3^, non-invasive prenatal test.

**Table 2:** Associations between fetal fraction and different parameters.

Parameters Associated with FF ^1^	Statistical Significance	Statistical Analysis/*p* Values
Gestational age	no significant correlation	Spearman Correlation ρ = 0.053/*p* = 0.418
Pregnant women in the age risk category, over 35 years	insignificantly lower	Mann-Whitney U test/*p* = 0.400
Maternal age	no significant correlation	Spearman Correlation ρ = −0.022/*p* = 0.740
Pregnant women who had the NIPT normal result	insignificantly increased	Mann-Whitney U test/*p* = 0.728
Pregnant women with no risk of aneuploidy	insignificantly increased	Kruskal-Wallis test/*p* = 0.315
Pregnant women at risk of microdeletion as a single abnormality	insignificantly increased	Kruskal-Wallis test/*p* = 0.176
Cases confirmed by invasive methods	insignificant association	Kruskal-Wallis test/*p* = 0.490

FF ^1^, Fetal fraction (%).

**Table 3:** Mean maternal age in different groups.

Maternal Age (Years)	Mean ± SD ^1^	Minimum–Maximum	Number of Cases
Group up to 34 years	29.8 ± 2.87	21–34	216
Group over 35 years	38.2 ± 2.61	35–47	164
Cases with NIPT risk for trisomy 21	34.8 ± 6.38	25–42	5
Cases with NIPT risk for trisomy 18	36.3 ± 7.10	30–44	3
Cases with NIPT risk for trisomy 13	39	39	1
Cases with NIPT risk for trisomy 14	41	41	1
Cases with NIPT risk for X monosomy	33.5 ± 4.95	30–37	2
Group with NIPT risk for all types of tested aneuploidy	35.83 ± 5.57	25–44	12
Group with confirmed aneuploidy	34.5 ± 5.21	25-42	8
Group with NIPT risk of aneuploidy and microdeletion	35.5 ± 6.09	26–41	6
Group with confirmed microdeletion	41	41	1
Entire group	33.4 ± 4.96	21–47	380

SD ^1^, standard deviation.

**Table 4:** The correlation between the risk assessed by NIPT and the final result.

NIPT Risk Assessment	Number of Cases	(%)	Invasive Prenatal Diagnosis			PPV ^1^ (%)
			Confirmed	False Positive	False Negative	
Total number of cases with NIPT result	378	100				
NIPT low risk	362	95.76	-	-	-	100
NIPT increased risk	16	4.23	8			
NIPT increased risk for isolated aneuploidies	10	2.64	7	3	-	70
Trisomy 21	5	1.32	5	0	0	100
Trisomy 18	3	0.79	2	1	0	66.66
Trisomy 13		0	0	0	1	0
Monosomy X	2	0.53	0	2	0	0
NIPT increased risk for aneuploidy and microdeletion	2	0.53	1	1	0	16.66
Trisomy 13 and 1p36 microdeletion syndrome	1	0.26	0 0	1 1	0	0
Trisomy 14 and del(X)(p22.33-11.21)	1	0.26	0 1	1 0	0	0
NIPT increased risk for isolated microdeletions	4	1.04	0	4	0	0
del(10q25.2-q26.3)	1	0.26	0	1	0	0
del(15)(q11.2-13.1)	1	0.26	0	1	0	0
del(20q11.21-q13.13)	1	0.26	0	1	0	0
del(22q11.2) microdeletion syndrome	1	0.26	0	1	0	0

PPV ^1^, Positive predictive value.

**Table 5:** Description of cases at risk for microdeletions.

NIPT High Risk for Microdeletions	Size (Mb ^1^) Determined by NIPT	FF ^2^ (%)	Gestational Age (Weeks)	aCGH ^3^ and Size (Mb)
del(X)(p22.33–11.21)	54.27	15.64	13	arr[GRCh37] Xp22.33p11.1 (168551_62051248)x1 61.88 Mb
del(10q25.2–q26.3)	22.57	5.3	11	False-positive
del(20q11.21–q13.13)	18.40	8.32	12	False-positive
del(15)(q11.2–13.1)	5.60	10.96	13	False-positive
del(22q11.2) microdeletion syndrome	2.54	9	12	False-positive
1p36 microdeletion syndrome	2.30	12.3	16	False-positive

Mb ^1^, Megabase; FF ^2^, fetal fraction; aCGH ^3^, Array CGH.

**Table 6:** Comparison of specificity, sensitivity and ppv scores for aneuploidies between NIFTY and NIPS tests.

Chromosomal Abnormality	Sensitivity		Specificity		PPV ^1^		
	NIFTY BGI	NIPS INVITAE	NIFTY BGI	NIPS INVITAE	NIFTY BGI	NIPS INVITAE	Present study
Trisomy 21	99.17%	99.99%	99.95%	99.89%	92.19%	92.89	100%
Trisomy 18	98.24%,	99.99%	99.95%	99.99%	76.61%	89.11	66.66%
Trisomy 13	>99.9%	99.99%	99.96%	99.69%	32.84%	73.54	0%
Monosomy X	>99.9%	99.99%	-	99.89%	40%	69.15	0%

PPV ^1^, Positive predictive value.

## Supplementary Materials

The following are available online at https://www.mdpi.com/article/10.3390/medicina58010079/s1, Table S1: Combinations of indications and the results of the study.
