# Supplementary material for: Genetic Counseling and Management: The First Study to Report NIPT Findings in a Romanian Population
Source: Medicina (Kaunas). 2022 Jan 5;58(1):79. doi: 10.3390/medicina58010079 (PMC8777823; doi:10.3390/medicina58010079)
Supplement: Supplementary file 1 [file medicina-58-00079-s001.zip › medicina-1506011-supplementary.pdf]

Table S1. Combinations of indications and the results of the study.

Combinations of Indications	Indication 1	Indication 2	Indication 3	Indication 4	NIPT result	Invasive Test Result: Final Result
Maternal age (years) + Ultrasound fetal abnormalities	Maternal age 37	Ultrasound: Tricuspid valve regurgitation			Low risk	Not necessary
	Maternal age 38	Ultrasound: renal pyelectasis, sexual ambiguity.			Low risk	Not necessary
	Maternal age 37	Ultrasound: Pre-testing cystic hygroma with suspicion of Turner syndrome. Post- Nifty test, a second ultrasonography revealed polydactyly and heart abnormality.			High risk for monosomy X	CVS with QF-PCR: Trisomy 13
DT + Ultrasound fetal abnormalities	DT: 1/196 for Trisomy 21	Ultrasound: NT = 3mm			Low risk	Not necessary
	Increased AFP in maternal serum	Ultrasound: Intrauterine growth retardation, banana-shaped cerebellum, risk of neural tube defect			Low risk	Not necessary
DT + maternal age (years)	DT: 1/80 for Trisomy 21	Maternal age 36			Low risk	Not necessary
DT + positive obstetrical history	DT: 1/390 for Trisomy 18	History of hydrops fetalis with intrauterine fetal death			Low risk	Not necessary
DT + Chromosomal anomalies in the family	DT: 1/120 for Trisomy 21	The pregnant woman is a carrier of a translocation t(8q;16p)			Low risk	Not necessary
Maternal age (years) + Genetic abnormality in the family	Maternal age 39	The pregnant woman is a carrier of a large deletion extending from exon 48 to exon 51 in the dystrophin gene			Low risk Female fetus	Not necessary
	Maternal age 39	Partner with inversion of chromosome 9 inv(9)(p11;q13)			Low risk	Not necessary
Maternal age (years) + Genetic abnormality in the family + Positive obstetrical history	Maternal age 38	Previous child with 8p21.3→p23.3 duplication	Two miscarriages		Low risk	Not necessary
	Maternal age 44	Cousin with trisomy 21			High risk for trisomy 18	CVS with QF-PCR: normal
Maternal age (years) + DT + Ultrasound fetal abnormalities + Positive obstetrical history	Maternal age 41	DT: ¼ for Trisomy 21	Bilateral hyperechogenic kidney; hyperechog	Previous fetus with trisomy 21	High risk for trisomy 14 and del(X)	Deletion del(X) (p11.1-ter); confirmed Trisomy 14 was not confirmed

enic fetal bowel; abnormal venous duct, bilateral hydrothorax; ascites
